# Prevalence and Antimicrobial Resistance of Bacteria Isolated from Marine and Freshwater Fish in Tanzania

**DOI:** 10.1155/2022/4652326

**Published:** 2022-03-04

**Authors:** Esther Marijani

**Affiliations:** Open University of Tanzania, P.O. Box 23409, Dar es Salaam, Tanzania

## Abstract

This study aimed to determine the prevalence and antimicrobial resistance of bacteria isolated from retail fish and shrimp in Tanzania. A total of 92 fish and 20 shrimp samples were analyzed. Fish samples consisted of 24 Nile tilapia, 24 Nile perch, and 24 red snapper. The isolates were identified by their morphological characteristics, conventional biochemical tests, and analytical profile index test kits. The antibiotic susceptibility of selected bacteria was determined by the disc diffusion method. Out of the 92 samples analyzed, 96.7% were contaminated with 7 different bacterial species. *E. coli* was the most prevalent bacteria (39%), followed by *Klebsiella* spp. (28%) and *Salmonella* spp. (16%). Other species isolated from this study were *Staphylococcus* spp. (8%), *Citrobacter* (4%), *Shigella* spp. (3%), and *Pseudomona*s spp. (1%). All samples were analyzed for *Campylobacter* spp.; however, none of the samples tested were positive for *Campylobacter* spp. Fish from the open-air market were contaminated by six bacterial species: *E. coli* (40%), *Klebsiella* spp. (26%), *Salmonella* spp. (24%), *Shigella* spp. (6.7%), *Citrobacter* spp. (6.5%), and *Pseudomonas* spp. (2%), while *E. coli* (37%), *Klebsiella* spp. (33%), *Staphylococcus* spp. (23%), and *Shigella* spp. (2%) were isolated in supermarket samples. According to the International Commission on Microbiological Specifications for Foods criteria, 54 (58.7%) and 38 (41.3%) samples were good and marginally acceptable, respectively. *E. coli* isolates were resistant to penicillin (PEN), erythromycin (ERY), gentamicin (GEN), azithromycin (AZM), and tetracycline (TET), while *Salmonella* spp. isolates exhibited resistance to gentamicin (CN), tetracycline (TET), penicillin (PEN), and erythromycin (ERY). These results suggest that the presence of these bacteria might cause a health risk/hazard to human beings and may cause disease to susceptible individuals, especially immune-compromised consumers.

## 1. Introduction

Fish are a vital source of food for people and are the most important source of high-quality protein. Fish provide approximately 16% of the animal protein consumed by the world's population [1]. Approximately 60% of developing countries derive 30% of their annual protein from fish [2]. Fish provide 19% of protein and are one of the cheapest sources of protein in Africa. In Tanzania, per capita, fish consumption is 8.0 kilograms, and fish make up 19.7% of the country's animal protein intake [3]. However, fish consumption is much higher in coastal regions and Zanzibar (almost 20 kg per capita), as well as along the coasts of Lake Tanganyika and Lake Victoria. Apart from being a rich source of protein, fish also play a unique role in providing a range of micronutrients and essential fatty acids, especially long-chain polyunsaturated fatty acids, which cannot be easily substituted by other food commodities [4, 5]. Despite these numerous advantages of fish, they are susceptible to a wide variety of bacterial pathogens, most of which can cause disease and are considered by some to be saprophytic in nature [1]. Fish and fishery products are known to be sources of transmitting foodborne infections and intoxication if not properly handled/processed [6].

It has been reported that the microbiological diversity of fresh fish muscle depends on the fishing grounds and environmental factors around it [7]. According to Clucas and Ward [8], the type of microorganisms that are associated with a particular fish depends on their habitat.

Bacterial pathogens associated with fish are classified as indigenous and nonindigenous. Examples of nonindigenous bacterial pathogens include *Escherichia coli*, *Clostridium botulinum*, *Shigella dysenteriae*, *Staphylococcus aureus*, *Listeria monocytogenes,* and *Salmonella*. These nonindigenous organisms contaminate the fish or the habitat in one way or the other. Examples of indigenous bacterial pathogens are *Vibrio* species and *Aeromonas* species, which are found naturally living in fish habitats [9, 10].

The bacteria from fish only become pathogens when there are stressors, such as poor water quality and overstocking, which allow opportunistic bacterial infections to prevail [1]. Additionally, studies have reported the presence of indicator microorganisms of fecal contamination, opportunistic, and pathogenic bacteria to humans in fish samples [11, 12].

Human infections caused by pathogens transmitted from fish are widespread and depend on the season, the patients' contact with fish and related environments, dietary habits, and the immune system status of the individual [13]. Pathogens can be transmitted through food or the handling of fish. Foodborne illnesses such as dysentery and diarrhea resulting from the consumption of contaminated fish can result in economic losses. Microbial associations with fish compromise the safety and quality of human consumption; this is particularly critical when microorganisms are opportunistic and/or pathogenic in nature [11, 13].

Poor fish handling, processing, and packaging methods and practices in retail markets are still common in Tanzania [14]. There are limited studies on the prevalence of bacteria isolated from retail fish and shrimps in Tanzania. The risk of getting foodborne diseases or food poisoning by fish consumers and handlers may be high in Tanzania. Therefore, this study investigates the prevalence of bacteria isolated from retail fish and shrimps in Tanzania.

Several antibiotics used in agriculture and aquaculture are critical for human medicine by the World Health Organization (WHO), including the antibiotic classes of tetracyclines, quinolones, and penicillin [15].

Resistance to all antibiotic classes has been observed in a wide range of bacteria, including those pathogenic to humans [16, 17].

Antimicrobial resistance is widespread in Tanzania, and there is widespread misuse of antimicrobials in the livestock and aquaculture industries. However, there is a scarcity of information regarding antibiotic resistance in Tanzania [18]. The scarcity of data may have contributed to the use of drugs without a prescription or prescribing drugs without carrying out a laboratory test to identify the pathogens and test their susceptibility to drugs for effective treatment.

Hence, another goal of this study was to determine the prevalence and antimicrobial resistance of foodborne pathogens isolated from retail fish and shrimps in Tanzania.

## 2. Methodology

### 2.1. Sample Collection

A total of 92 fresh fish samples of both marine and freshwater were purchased from two different open-air fish markets (*n* = 60) and two supermarkets (*n* = 32) in the Ilala district, Dar es Salaam, Tanzania. Out of the 92 samples collected, twenty-four samples were of Nile tilapia, Nile perch, red snapper, and twenty shrimp. After the collection, samples were aseptically and immediately transported in a sterile polypropylene bag placed in a cooler box. The cooler box contained crushed ice, and the temperatures were between 4°C and 8°C during transportation. The samples were transported to the Open University Laboratory, and for further analysis, the samples were analyzed at the College of Veterinary and Biomedical Sciences, Sokoine University of Agriculture.

### 2.2. Culture, Isolation, and Identification

For isolation and identification of bacteria, culturing was performed by chopping a piece of fish flesh aseptically and spreading it over the surface of blood agar and MacConkey agar before incubation between 24 and 48 hours at 37°C. For *Campylobacter* spp. identification, a piece of fish samples was first enriched in Bolton broth overnight and later inoculated onto modified charcoal cefoperazone deoxycholate agar (mCCDA) (Oxoid Ltd., Basingstoke, Hampshire, England) containing the *Campylobacter* mCCDA selective supplement SR155 E (Oxoid Ltd., Basingstoke, Hampshire, England). Incubation was performed as previously described by Kurekci et al. [19] at 37°C for 48 h under microaerophilic conditions generated by CampyGenTM gas sachets (Oxoid, Basingstoke, England, UK).

Gram staining was done to differentiate organisms based on the structure of their cell walls [20]. Furthermore, the classical identification of bacterial colonies and biotyping were performed according to the methods described by Abbott et al. [20] and El Deen et al. [21] with slight modifications. Briefly, the isolates were conventionally studied for their macro- and micromorphological characteristics and then by biochemical assays that consisted of 21 phenotypic characteristics tests. The assays included lactose, raffinose, trehalose, dulcitol, maltose, mannose, D-mannitol, melibiose, sucrose, citrate, urea, indole, catalase, motility, ampicillin resistance, m-inositol, oxidase, nitrate, cellobiose, and xylose. Triple sugar iron agar and IMViC were also used for the characterization of members of the family Enterobacteriaceae.

### 2.3. Microbial Load by Total Viable Count

To assess the level of microbial contamination, 10 g of fish pieces was rinsed in 10 mL of sterile normal saline. Then, 1 mL of the rinse was serially diluted 10-fold using 10 universal bottles containing sterile normal saline. In each dilution, 1 mL was poured on the plate count of medium Petri dishes in duplicate. The plates were incubated at 37°C for 24 h. Then, colonies were counted, and the average colony counts were used to establish the colony-forming units (CFU/mL or CFU/g).

### 2.4. Antimicrobial Assay

Antimicrobial susceptibility testing was performed using the disc diffusion method. The isolates were tested against quinolones, namely, ciprofloxacin (CIP); macrolides, erythromycin (E), and azithromycin (AZM); and aminoglycosides, gentamicin (CN) (0.06–64 *µ*g/mL), and tetracycline (TE). Other antimicrobials were clindamycin (DA) and penicillin (P). All antimicrobials were supplied by Sigma-Aldrich (St. Louis, MO, USA). For antimicrobial susceptibility assays, a pool of bacterial colonies was used to prepare suspensions corresponding to 0.5 McFarland standards (1.5 × 10^8^ CFU/mL) using normal saline, and then bacteria were spread on top of Müller-Hinton agar using a sterile swab. Discs were placed on top of the medium, and the plates were then incubated at 37°C for 24 h. Zones of inhibition were measured by means of a simple ruler, and the diameter was recorded in millimeters (mm).

Isolates were defined as susceptible, intermediate, or resistant in accordance with the CLSI [22] *Enterobacteriaceae* breakpoints.

### 2.5. Data Analysis

Data from counts (in log CFU/g) were analyzed (means, standard deviations, and standard errors) using the Statistical Package SPSS v21 (IBM Corporation, Armonk, NY, USA). Descriptive statistics were used to describe the results of the prevalence analysis. The prevalence was estimated as the number of samples detected positive for *Salmonella* spp. *Campylobacter* spp., *E. coli*, *Shigella* spp., and *Staphylococcus* spp. isolation from the total sample analyzed.

## 3. Results

### 3.1. Total Viable Count (TVC) and Microbiological Quality Data

The average TVC for the 92 samples was 4.57 log CFU/g, ranging from 3.82 to 4.70 and 5.22 log CFU/g in tilapia, Nile perch, and red snapper, respectively. A significant difference (*P* < 0.05) was observed between Nile tilapia and red snapper samples, as shown in Table 1. When comparing total samples (Table 1), red snapper had the highest mean TVC at 5.22 log CFU/g, and Nile tilapia had the lowest at 3.82 log CFU/g (*P* < 0.05). There is no significant difference (*P* > 0.05) in TVC log CFU/g between samples from the supermarket and retail market. However, samples from the supermarket have a lower TVC at 3.77 log CFU/g compared to samples from the open-air market (4.99 log CFU/g) (data not shown).

Based on the International Commission on Microbiological Specifications for Foods criteria, 54 (58.7%) and 38 (41.3%) samples were good and marginally acceptable, respectively (Table 1). Nile tilapia had a higher good quality sample, followed by shrimp, Nile perch, and red snapper. None of the samples had unacceptable microbiological quality.

### 3.2. Prevalence of *Campylobacter*, *E. coli*, *Listeria*, *Salmonella*, and *Shigella*

The prevalence and contamination rates of open-air market fish with pathogenic bacteria are presented in Table 2). Seven bacterial species were identified from all analyzed samples. The most prevalent bacterial species recovered in the samples was *E. coli* (39%), followed by *Klebsiella* spp. (28%) and *Salmonella* spp. (16%). Other species isolated from this study were *Staphylococcus* spp. (8%), *Citrobacter* (4%), *Shigella* spp. (3%), and *Pseudomona*s spp. (1%). Nile tilapia were highly contaminated by *E. coli* (48%), followed by shrimp (45%). Nile perch were highly contaminated by *Salmonella* spp., followed by Nile tilapia. Only shrimp and Nile perch were contaminated by *Shigella* spp., while *Staphylococcus* spp. were isolated in Nile tilapia and Nile perch only.

All samples were screened for *Campylobacter*, but no *Campylobacter* was recovered by culture.

**Figure 1:**
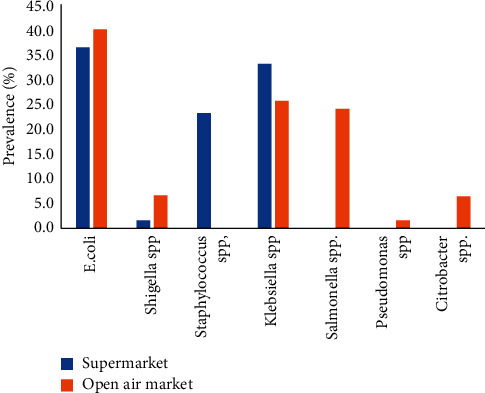
Prevalence of different bacteria on fish from open-air markets and supermarkets.

Samples from the open-air market were contaminated by six bacterial species, whereas four species were detected in supermarket samples (Figure 1). Fish from the open-air market were contaminated by *E. coli* (40%), *Klebsiella* spp. (26%), and *Salmonella* spp. (24%) while supermarket samples were contaminated by *E. coli* (37%), *Klebsiella* spp. (33%), and *Staphylococcus* spp. (23%). *Salmonella* spp. was not detected in the supermarket samples, while *Staphylococcus* spp. was not detected in the open-air market samples.

### 3.3. Antibiogram Profile of Isolated Bacteria

In general, the isolates showed a low level of resistance to most of the antimicrobial agents. Of the 48 *E. coli* isolates, 14 (29%) were resistant to penicillin, 12 (25%) were resistant to erythromycin, 8 (17%) were resistant to gentamicin, and 7 (15%) and 5 (10%) were resistant to azithromycin and ciprofloxacin, respectively. For *Salmonella* spp. isolates, 8 (31%), 7 (27%), 6 (23%), and 3 (12%) showed resistance to gentamicin, tetracycline, penicillin, and erythromycin, respectively.

“For *Staphylococcus* spp., among the 16 isolates, 31% and 5% were resistant to tetracycline and gentamicin, respectively. However, resistance to ciprofloxacin, clindamycin, and penicillin was observed in 19%, 13%, and 13% of isolates, respectively.

*Klebsiella* spp. exhibited resistance to six antimicrobial agents comprising gentamicin, tetracycline, penicillin, erythromycin, azithromycin, and ciprofloxacin, whereas more than 20% were resistant to gentamicin (Table 3).

**Figure 2:**
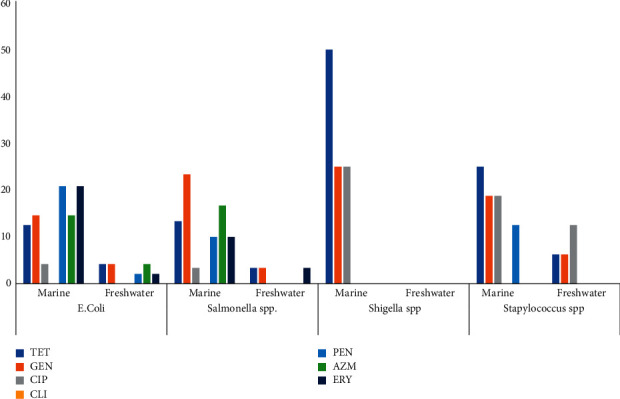
Prevalence of bacterial spp. resistance to antibiotics isolated from open-air markets and supermarkets. ^*∗*^TET = tetracycline; GEN = gentamicin; CIP = ciprofloxacin; CLI = clindamycin; PEN = penicillin; AZM = azithromycin; ERY = erythromycin.

As observed in Figure 2, bacterial isolates from marine fish were more resistant to antibiotics than those isolated from freshwater fish. *Shigella* spp. isolated from marine fish were resistant to tetracycline 28 (17%), gentamicin, and ciprofloxacin, while no isolates from freshwater were resistant to antibiotics.

*Salmonella* spp. isolates recovered from marine fish showed resistance to six antimicrobial agents comprising gentamicin, tetracycline, penicillin, erythromycin, azithromycin, and ciprofloxacin. *Salmonella* spp. isolates recovered from freshwater fish exhibited resistance to only three antimicrobial agents comprising gentamicin, tetracycline, and erythromycin.

## 4. Discussion

Most outbreaks of food poisoning related to fish are derived from the consumption of raw or insufficiently heat-treated fish and cross-contamination during handling. According to Huss et al. [24] and Aberoumand [25], approximately 12% of foodborne outbreaks related to the consumption of fish are caused by bacteria.

In this study, a total of 7 different species of bacteria were isolated. The most prevalent bacteria were *E. coli* (39%), followed by *Klebsiella* spp. (28%) and *Salmonella* spp. (16%). Other bacteria isolated in this study are *Staphylococcus* spp. (8%) and *Shigella* spp. (3%). Citrobacter (4%) and *Pseudomona*s spp. (1%) were also isolated in this study.

*Salmonella* spp. was detected in fish samples (16%) collected from different retail markets in this study. Higher results were reported by Kumar et al. [26], Jegadeeshkumar et al. [27], and Budiati et al. [28] at rates of 30%, 43.8%, and 90% in fish samples, respectively. In contrast, other studies from Trinidad and Turkey detected *Salmonella* spp. at 0% and 5% in fish samples, respectively [29, 30].

The presence of some bacterial species isolated from this study can cause food poisoning, diarrhea, typhoid fever, and shigellosis. Sichewo et al. [1] suggested that, when present in food, pathogens such as *S. aureus*, *Salmonella*, *Shigella*, and *Pseudomonas* are most likely to cause foodborne diseases. The presence of *Salmonella*, *Shigella*, and *E. coli* in fish indicates faecal and environmental contamination [1]. Coliforms such as *E. coli* are not normal bacteria in fish and are commonly present where there has been faecal contamination from warm-blooded animals [31]. *E. coli* is known to be a reliable indicator of fecal contamination in small numbers and large numbers, and it is an indicator of mishandling [32]. The presence of *E. coli* in this study could be attributed to poor mishandling of fish by traders. The presence of these bacteria isolated from the open-air market samples might also possibly be due to unhygienic handling during transportation and storage. The use of contaminated water for cleaning and processing fish in the fish market is presumably the cause of secondary contamination. The lack of proper drainage facilities and heavy fly infestation in these fish markets also promotes tertiary contamination to a great extent.

The isolation rate of *Staphylococcus* spp. in the retail fish samples in the present study (8%) is relatively low compared with previous reports, which had rates ranging from 19.9% to 61.7% [33–36]. However, in this study, *Staphylococcus* spp. were isolated in samples from supermarkets only, which might be associated with inappropriate handling of fish.

The findings of this study show that the mean TVC for 92 samples was 4.57 log CFU/g, ranging from 3.82 to 4.70 and 5.22 log CFU/g in Nile tilapia, Nile perch, and red snapper, respectively. This is similar to the study of Wang et al. [37], who found the TVCs for salmon and tilapia to be 5.58 and 4.37 log CFU/g, respectively. According to the International Commission on Microbiological Specifications for Foods criteria, the microbiological quality of the fish examined in this study is acceptable and does not pose a potential risk to public health.

The results from this study also showed that the enteric bacteria isolated from fish were resistant to some antibiotics. It was observed that a small percentage of *Salmonella* spp. were resistant to gentamicin, tetracycline, penicillin, and erythromycin. Similar to our results (except erythromycin), Ponce et al. [38] found that the majority of isolates were resistant to ampicillin, tetracycline, chloramphenicol, gentamicin, sulfisoxazole, streptomycin, and kanamycin.

A total of 29, 25, 17, 15, and 13% of the *E. coli* isolates exhibited resistance to penicillin, erythromycin, gentamicin, azithromycin, and tetracycline, respectively. This is in contrast to the results obtained by Kibret and Abera [39], where erythromycin and tetracycline were highly resistant to *E. coli* isolates at 89.4 and 72.6%, respectively. The high prevalence of resistance to tetracycline, ampicillin, and cotrimoxazole *in E. coli* in the region has also been reported by Sifuna et al. [40], in which *E. coli* demonstrated resistance mostly to ampicillin and tetracycline. According to Sifuna et al. [40], the resistance pattern reported in their study can also be linked to the use of these drugs in veterinary practice and lead to resistance in humans.

In this study, bacteria isolated from marine fish were more resistant to antibiotics than those from freshwater fish. *Salmonella* spp. isolates recovered from marine samples were resistant to six antimicrobial agents, while isolates recovered from freshwater fish were resistant to three antimicrobial agents. *Shigella* isolates recovered from marine fish were resistant to tetracycline, gentamicin, and ciprofloxacin. However, *Shigella* isolates recovered from freshwater samples were not resistant to any antimicrobial agents. In contrast, a study on the antimicrobial resistance in isolates from marine species showed a high occurrence of antibiotic resistance, with 68% of isolates demonstrating resistance to at least one antibiotic, which is higher than the occurrence of antibiotic resistance observed in the current study [41]. Another study examining antimicrobial resistance in marine mammals reported *E.coli* was resistant to doxycycline (5%), amoxicillin (4%), and gentamicin (1%). Lockwood et al. [42] carried out a study on bacteria isolated from stranded harbor seals over a 12-year period. The author reported that bacterial isolates exhibit resistance most frequently to ampicillin (74% resistant) and cephalothin (64% resistant). Antibiotic-resistant microbes and genes within the aquatic environment have been documented in various marine species from cephalopods to marine mammals and elasmobranchs, such as sharks [43–47].

Rose et al. [41] and Ahmed et al. [48] concluded that the presence of antibiotic-resistant bacteria from animals, and marine animals, in particular, indicates not only the widespread presence of the microbes but often a significant percentage of the bacteria demonstrating resistance to multiple antibiotics.

Oates et al. [49] reported that when wastewater discharge and runoff from agricultural activity, which carries antibiotics and resistant bacteria into terrestrial waterways, finds its way to marine coastlines, it may cause disease in marine organisms, contributing to antibiotic resistance. This, along with other anthropogenic contributions, may contribute to the elevation of natural background levels of antibiotic resistance genes in aquatic environments, encouraging their transfer into pathogens or serving as a means for antibiotic resistance propagation [44]. When resistant bacteria are introduced to animals or their environment, the animals may become sick, or resistance traits may be transferred to other bacterial species, or they may become a reservoir that transfers the bacteria and resistance back to humans and the environment [45]. The antibiotics used by the majority of the Tanzanian population are unregulated and used indiscriminately, and statistics are rarely collected, hence increasing the risk to the environment and human and animal health. Therefore, due to the widespread acquisition of resistance, it is important that susceptibility tests be routinely performed to guide antibiotic treatment and policy.

## 5. Conclusion

In conclusion, this study demonstrated the presence of highly pathogenic agents such as *Salmonella* and *Shigella* species and opportunistic pathogens, and some carried antimicrobial resistance.

Their presence is a potential health risk/hazard to human beings and may cause disease to susceptible individuals, especially immune-compromised consumers.

This study has therefore proven the need for the adoption of proper hygienic measures from farm to fork and hygienic education for fish handlers/traders and consumers.

In addition, bacterial isolates recovered from marine fish samples were more resistant to antibiotics than isolates recovered from freshwater fish samples. The environment might be a reservoir of resistance genes and dispersal vectors due to the influence of anthropogenic activities in marine environments. The resistance patterns observed in this study imply that some components of resistance are likely related to environmental origins and may spread without the selective pressure of antibiotic use.

There is a need for research on antibiotic susceptibility surveillance in aquatic environments where fresh fish and marine fish are obtained for human consumption.

## Figures and Tables

**Table 1:** Mean total viable counts of retail fish in log10 CFU per gram ± standard error (SE).

Fish species	*N*	^*∗*^Mean ± SE	% of samples that fell into the quality category^*∗∗*^		
			Good	Marginally acceptable	Unacceptable
Nile perch	24	4.70 ± 0.10^a^	16.30	8.70	0
Shrimps	20	4.53 ± 0.29^a^	15.22	6.52	0
Nile tilapia	24	3.82 ± 0.41^b^	19.57	6.52	0
Red snapper	24	5.22 ± 0.13^a^	7.61	19.57	0
Total	92		58.70	41.30	0

^*∗*^Means followed by similar letters do not differ significantly (*P* < 0.05). ^*∗∗*^Microbiological quality categories of good (<5 × 105 CFU/g), marginally acceptable (5 × 105 to 1 × 107 CFU/g), and unacceptable (>1 × 107 CFU/g) [23].

**Table 2:** Occurrence of different bacteria on retail fish from Dar es Salaam.

Bacterial species	Prevalence (%)				
	Red snapper (*n* = 24)	Shrimps (*n* = 20)	Nile perch (*n* = 24)	Nile tilapia (*n* = 24)	Overall (*n* = 92)
*Citrobacter* spp.	1 (4)^*∗*^	2 (10)	1 (4)	0	4 (4)
*E. coli*	10 (40)	9 (45)	6 (26)	12 (48)	36 (39)
*Klebsiella* spp.	9 (36)	7 (35)	4 (17)	6 (24)	26 (28)
*Pseudomonas* spp.	1 (4)	0	0	0	1 (1)
*Salmonella* spp.	3 (12)	2 (10)	6 (26)	4 (24)	15 (16)
*Shigella* spp.	1 (4)	0	2 (9)	0	3 (3)
*Staphylococcus* spp.	0	0	4 (17)	3 (12)	7 (8)

^*∗*^Values in parentheses indicate the % incidence.

**Table 3:** Resistance profiles of bacterial spp. isolated from open-air markets and supermarkets.

^*∗*^Antibiotics	Disc conc. (ug)	% of resistant isolates						
		*E. coli*	*Klebsiella* spp.	*Shigella* spp.	*Salmonella* spp.	*Pseudomonas* spp.	*Citrobacter* spp.	*Stapylococcus* spp.
TET	30	4	16	50	27	0	22	31
GEN	10	17	28	25	31	0	17	25
CIP	5	10	10	25	8	0	11	19
CLI	2	0	0	0	0	0	0	13
PEN	10	29	18	0	23	0	11	13
AZM	15	15	16	0	0	0	22	0
ERY	15	25	12	0	12	0	17	0

^*∗*^Antibiotics: TET = tetracycline; GEN = gentamicin; CIP = ciprofloxacin; CLI = clindamycin; PEN = penicillin; AZM = azithromycin; ERY = erythromycin.

## Data Availability

Data will be available on request.
